# Incidence of type 2 diabetes, cardiovascular disease and chronic kidney disease in patients with multiple sclerosis initiating disease-modifying therapies: Retrospective cohort study using a frequentist model averaging statistical framework

**DOI:** 10.1371/journal.pone.0300708

**Published:** 2024-03-22

**Authors:** Alan J. M. Brnabic, Sarah E. Curtis, Joseph A. Johnston, Albert Lo, Anthony J. Zagar, Ilya Lipkovich, Zbigniew Kadziola, Megan H. Murray, Timothy Ryan

**Affiliations:** Eli Lilly and Company, Lilly Corporate Center, Indianapolis, IN, United States of America; Fondazione Don Carlo Gnocchi, ITALY

## Abstract

Researchers are increasingly using insights derived from large-scale, electronic healthcare data to inform drug development and provide human validation of novel treatment pathways and aid in drug repurposing/repositioning. The objective of this study was to determine whether treatment of patients with multiple sclerosis with dimethyl fumarate, an activator of the nuclear factor erythroid 2-related factor 2 (Nrf2) pathway, results in a change in incidence of type 2 diabetes and its complications. This retrospective cohort study used administrative claims data to derive four cohorts of adults with multiple sclerosis initiating dimethyl fumarate, teriflunomide, glatiramer acetate or fingolimod between January 2013 and December 2018. A causal inference frequentist model averaging framework based on machine learning was used to compare the time to first occurrence of a composite endpoint of type 2 diabetes, cardiovascular disease or chronic kidney disease, as well as each individual outcome, across the four treatment cohorts. There was a statistically significantly lower risk of incidence for dimethyl fumarate versus teriflunomide for the composite endpoint (restricted hazard ratio [95% confidence interval] 0.70 [0.55, 0.90]) and type 2 diabetes (0.65 [0.49, 0.98]), myocardial infarction (0.59 [0.35, 0.97]) and chronic kidney disease (0.52 [0.28, 0.86]). No differences for other individual outcomes or for dimethyl fumarate versus the other two cohorts were observed. This study effectively demonstrated the use of an innovative statistical methodology to test a clinical hypothesis using real-world data to perform early target validation for drug discovery. Although there was a trend among patients treated with dimethyl fumarate towards a decreased incidence of type 2 diabetes, cardiovascular disease and chronic kidney disease relative to other disease-modifying therapies–which was statistically significant for the comparison with teriflunomide–this study did not definitively support the hypothesis that Nrf2 activation provided additional metabolic disease benefit in patients with multiple sclerosis.

## Introduction

Researchers are increasingly using insights derived from large-scale, standardised electronic healthcare data to inform drug development, identify and provide human validation of novel treatment pathways and aid in drug repurposing and repositioning [1–7]. For example, in one such study, investigators used two large medical centre electronic health records in the United States (US) to validate the hypothesis that metformin, a first-line therapy for type 2 diabetes (T2D), was associated with decreased mortality after a cancer diagnosis compared with cancer patients not on metformin, indicating its potential as a chemotherapeutic regimen [3]. Another study used large volumes of data across four US insurance claims databases to identify medications associated with a ≥50% reduction in the risk of dementia and examined their biological pathways as targets for further research to aid in discovering novel therapeutic approaches to treating dementia [4].

The transcription factor Nrf2 (nuclear factor erythroid 2-related factor 2) is a master regulator of stress defence in the human body, as it orchestrates homeostatic adaptive responses to environmental or endogenous deviations in redox metabolism, proteostasis and inflammation [8, 9]. Preclinical studies have indicated that pharmacological activation of Nrf2 may be a promising therapeutic approach for several chronic diseases associated with high levels of oxidative stress and inflammation, such as T2D, chronic kidney, cardiovascular and other metabolic diseases [8–11]. Dimethyl fumarate, an activator of the Nrf2 pathway, is a Food and Drug Administration- and European Medicines Agency-approved first-line oral therapy for patients with relapsing forms of multiple sclerosis (MS) [12]. As dimethyl fumarate has been shown to be a Nrf2 activator in preclinical disease models, we sought to provide human evidence that modulating this target in patients taking dimethyl fumarate for MS might reduce the incidence of chronic diseases where Nrf2 activity has been implicated.

The therapeutic mechanism of action of dimethyl fumarate is not fully understood, but it is a di-methyl ester that, upon hydrolysis, targets reactive cysteine residues on specific proteins that sense oxidative stress [11]. One such protein with cysteine residues capable of sensing redox changes is Keap1, a Nrf2 binding partner that orchestrates adaptation to stress. Based on the adaptive changes invoked, this coordinated modulation of signalling through Nrf2/Keap1 would be expected to produce a prolonged protective response in concomitant diseases where oxidative stress has been implicated relative to other disease-modifying MS treatments not acting on this pathway, including fingolimod, teriflunomide and glatiramer acetate [11].

Our clinical hypothesis was that dimethyl fumarate, through its effect on Nrf2, may reduce the incidence of T2D, cardiovascular disease (CVD) and chronic kidney disease (CKD), in patients with MS. Although there are many studies comparing the rates of comorbidities in people with and without MS, there are no real-world studies comparing the incidence of T2D, CVD and CKD in patients with MS by their use of disease-modifying therapies (DMTs). We aimed to investigate whether this hypothesis could be supported by real-world evidence from administrative claims databases by applying comparative analysis in a causal inference frequentist model averaging (FMA) [13–15] framework based on machine learning. Our objective was to perform an early target validation of the effects of a Nrf2 pathway activator, dimethyl fumarate, on the incidence of chronic diseases (T2D, CVD and CKD) in human populations prior to embarking on lengthy preclinical drug development initiatives in related indications. In addition to providing human target validation, positive findings from such a study could serve to identify the most promising clinical indication, thus motivating future development of new therapies for these indications that work through the Nrf2 pathway.

## Materials and methods

### General

#### Objectives

The primary objective of this analysis was to report the incidence and compare the time to first occurrence of a composite endpoint of T2D, CVD (defined as acute heart failure [AHF], atherosclerosis, myocardial infarction [MI] or stroke) or CKD in patients with MS initiating either dimethyl fumarate, fingolimod, glatiramer acetate or teriflunomide. We also examined the incidence and time to first occurrence of each outcome (i.e., T2D, CVD, AHF, atherosclerosis, MI, stroke and CKD) individually across the four treatment groups as an exploratory objective. As a sensitivity analysis, we examined the composite endpoint and individual outcomes from index to drug discontinuation.

#### Data source

This retrospective cohort study used individual patient-level, de-identified, healthcare claims from the Merative L.P.^®^ Commercial and Medicare Supplemental Databases 2012 to 2019, which are fully compliant with US privacy laws and regulations. These data include health insurance claims across the continuum of care (e.g., inpatient, outpatient, outpatient pharmacy and carve-out behavioural healthcare), as well as enrolment data from large employers and health plans across the US who provide private healthcare coverage for more than 120 million employees, their spouses and dependents. These databases include a variety of fee-for-service, preferred provider organisation and capitated health plans.

All database records are de-identified and fully compliant with United States patient confidentiality requirements, including the Health Insurance Portability and Accountability Act (HIPAA) of 1996. The databases have been evaluated and certified by an independent third party to be in compliance with the HIPAA statistical de-identification standard. The databases were certified to satisfy the conditions set forth in Sections 164.514 (a)-(b)1ii of the HIPAA privacy rule regarding the determination and documentation of statistically de-identified data. Because this study uses only de-identified patient records and does not involve the collection, use, or transmittal of individually identifiable data, the data does not involve human subjects (per the definition of human subjects in the Code of Federal Regulations (CFR) Title 45 Part 46.102(e)). Thus, this study was exempted from Institutional Review Board (IRB) approval. Data was used under license for this study.

#### Study design

The retrospective study index, pre- and post-index and follow-up periods are shown in S1 Fig in **Supporting information**. The index period was from 01 January 2013 to 31 December 2018, with pre- and post-index periods of 1 year each, including the index day. Patients were followed from the index date until insurance disenrollment (i.e., a gap in continuous enrolment of >90 days) or the end of the study database period (31 December 2019).

#### Study population

Four cohorts of adults with MS initiating DMTs were identified based on their first DMT prescription during the index period: the dimethyl fumarate, fingolimod, glatiramer acetate and teriflunomide cohorts. Fingolimod, glatiramer acetate and teriflunomide (all small molecules) were selected for inclusion in the analysis, as control comparisons to dimethyl fumarate, based on their use in similar patient populations at similar stages of disease progression and available sample size from the chosen data source.

Eligible patients had at least one prescription for a DMT (dimethyl fumarate, fingolimod, glatiramer acetate or teriflunomide) during the index period. The index date was defined as the first DMT prescription during the index period and the index medication cohort was assigned based on the medication filled on the index date. Furthermore, the proportion of days covered (PDC) for the index drug had to be ≥60% during the 1-year post-index period, to ensure patients continued to fill their index drug prescription, and continuous enrolment with medical and pharmacy benefits was required during the pre-index period through the 1-year post-index period, although a 30-day gap was allowed. At least two diagnoses for MS were required during the pre-index period and patients had to be ≥18 years of age at index. Patients with diagnoses for the outcomes of interest or prescriptions for the index or comparator DMTs during the pre-index period were excluded. This ensured that only new initiators were included in the analysis and patients with prevalent disease at index were excluded.

#### Study measures

The primary composite endpoint was defined as incident T2D, CVD or CKD. T2D was defined as at least two diagnosis codes for T2D or at least one T2D diagnosis and at least one prescription for a diabetes medication. CVD was defined as a diagnosis for AHF, atherosclerosis, MI, or stroke. CKD was defined as at least one diagnosis for CKD. The codes used to define the outcomes are available in the **Supporting information** (S1 Table).

Patient demographics assessed at the index date included age, sex, region of residence, index year and payer type. Baseline clinical characteristics assessed during the pre-index period were hypertension, hyperlipidaemia, tobacco use, use of mobility aids (as a proxy for MS severity) and chronic disease burden using the Charlson Comorbidity Index. Duration of patients’ follow-up period, discontinuation of index drug and time to drug discontinuation were also examined.

### Statistical methods

#### Descriptive analyses

Comparisons of baseline characteristics and follow-up variables were conducted across cohorts using ANOVA or Mood’s median test for continuous variables and Fisher’s exact test or Monte Carlo Fisher’s exact test for categorical variables. Time-to-event (TTE) analyses, time to first occurrence of the composite endpoint, were reported descriptively using unadjusted Kaplan–Meier estimates for the four DMT cohorts. Patients discontinuing the study were censored from their last available values onward (i.e., when lost to follow-up or after death). For the TTE sensitivity analysis, patients were censored at the time of treatment discontinuation. Censoring rates were similar among treatment groups.

#### Comparative analyses–adjustments for bias and confounding

Comparisons between groups were conducted using an FMA approach to combine multiple analysis strategies (treatment and/or outcome models) to obtain a more accurate estimate of the treatment effect [13]. The analysis strategies attempt to adjust for imbalances found at baseline between treatment groups due to the non-randomised study design. Causal average treatment effect (ATE) estimators for comparing incidence and time to first occurrence of T2D, CVD, CKD and other outcomes across the four cohorts of interest were evaluated. The FMA methodology recently proposed by Zagar et al. [13] in the context of continuous outcomes was used. This methodology was adapted for use with binary and time to event (TTE) outcomes and applied to evaluate the ATE (defined in terms of restricted mean survival time [RMST] and restricted hazard ratio [rHR]) for each distinct pair of treatment cohorts.

**Fig 1:**
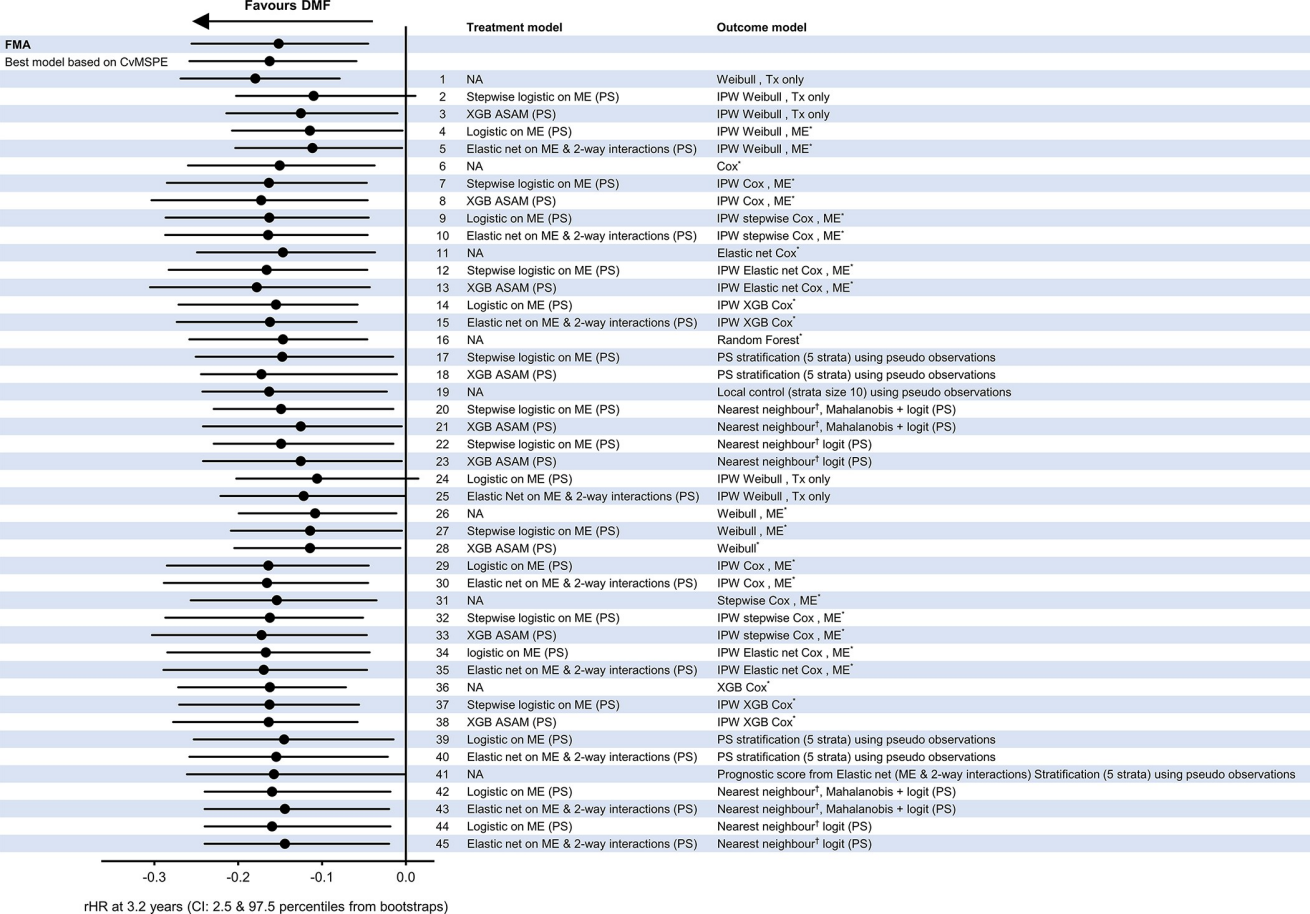
Forest plot of model combinations used for dimethyl fumarate versus teriflunomide primary composite endpoint comparison. Time to event analysis of DMF versus teriflunomide cohorts compared for the primary composite endpoint. *Separate models by treatment arm, ^†^e.g., matching, stratification. ASAM, average standardised absolute mean difference; ATE, average treatment effect; Cox, Cox proportional hazards regression model; CI, confidence interval; CvMSPE, cross-validated mean square prediction error; DMF, dimethyl fumarate; FMA, frequentist model averaging; IPW, inverse probability weighting; ME, main effects; NA, not applicable; PS, propensity score; rHR, restricted hazard ratio; Tx, treatment; XGB, extreme gradient boosting.

FMA combines ATE estimates from a broad set of estimators, incorporating available pre-treatment covariates to adjust for potential bias due to confounding, including methods based on direct covariate adjusted regression, inverse probability of treatment weighting, stratification and matching. The model strategies used in this FMA included treatment models that were used to balance the treatment cohorts, which were calculated using either logistic regression (stepwise or penalised), random forests or gradient boosting models [16]. The index variables (i.e., patient characteristics) used in the balancing scores are listed above under **Study measures**. To assess whether balance was achieved, the standardised difference (acceptable range <0.25) [17] and variance ratio (acceptable ranges 0.5–2.0) statistics were assessed (as per Austin et al. 2009 [18]). Outcome models included parametric and semi-parametric (Cox regression model) survival models fitted with and without penalisation. These models were implemented with the main effects as well as all two-way interactions. Random forests, gradient boosting and stratification (based on propensity score) were also considered as outcome models. The set of methods (individual model strategies) used in computing the FMA for this study are provided in Fig 1.

The odds ratio was the estimand of interest for incidence rates (a binary outcome) and the difference in the RMST or the rHR at 3.2 years TTE was the estimand for TTE outcomes [19, 20]. Note, the 3.2-year cut-off was chosen as this was the mean and median follow-up time for the primary objective.

The FMA estimates were computed as weighted averages of analysis strategies’ estimates with weights reflecting the level of support of the data for each individual strategy, based on cross-validated mean squared prediction error (CvMSPE). Larger values of CvMSPE indicate poorer support for a given strategy from the data and translate into a smaller weight for that strategy in the FMA estimator. Specifically, the *CvMSPE(S)* under individual strategy *S* for comparing two treatment cohorts T = {0/1} of sizes N_0_, N_1_, N = N_0_+N_1_, with individual patients in the two cohorts referred to by *i* = 1,..,*N*_0_ and *i* = 1+*N*_0_,..,*N*, respectively, is computed as:

$$CVMSPE(S)={\left(N_{0}+N_{1}\right)}^{-1}\left[\sum_{i=1}^{N_{0}}{{(Y_{i}-\hat{m}}_{S}^{-i}(0,x_{i}))}^{2}+\sum_{i=1+N_{0}}^{N}{\left(Y_{i}-{\hat{m}}_{S}^{-i}(1,x_{i})\right)}^{2}\right],$$

where *Y*_*i*_ is the observed outcome for the *i*th patient; ${\hat{m}}_{S}^{-i}(0,x_{i})$ and ${\hat{m}}_{S}^{-i}(1,x_{i})$ are predicted outcomes for that patient under strategy *S*, given his/her pre-treatment covariates *x*_*i*_ and assuming that the patient received (factually or contrary to fact) treatment *T* = 0 or *T* = 1, respectively. The superscript −*i* in ${\hat{m}}_{S}^{-i}$ indicates that the prediction is obtained by using cross-validation, that is with the data on the *i*th patients excluded from modelling the outcome and propensity functions. Further details can be found in Zagar et al. [13].

When the outcome is TTE, *CvMSPE*(*S*) is computed by replacing the observed survival outcomes *Y*_*i*_ with *pseudo-observations* introduced in the context of RMST, as shown in Andersen et al. [21]. The approach of Binder et al. [22] was used, which incorporates baseline covariates for computing pseudo-observations, as recommended in Andersen et al. [23]. The pseudo-observations for rHR were constructed similarly to those based on RMST by simply replacing the area underestimated survival distribution $\hat{S}(t)$ up to a cut-off time *t*_0_ with the ${-log(\hat{S}(t}_{0})$).

Region was the only covariate with missing information (2.0%) and was imputed as South (the most frequent region). Results presented from the FMA are rHR at 3.2 years with associated 95% confidence intervals (CI), computed using percentile bootstrapping, which Zagar et al. [13] showed provided appropriate coverage in the case of continuous outcomes. All analyses were conducted using SAS 9.4 (SAS Institute, Cary, NC, USA) and R Version 4.0.3 (R Foundation for Statistical Computing, Vienna, Austria).

## Results

**Fig 2:**
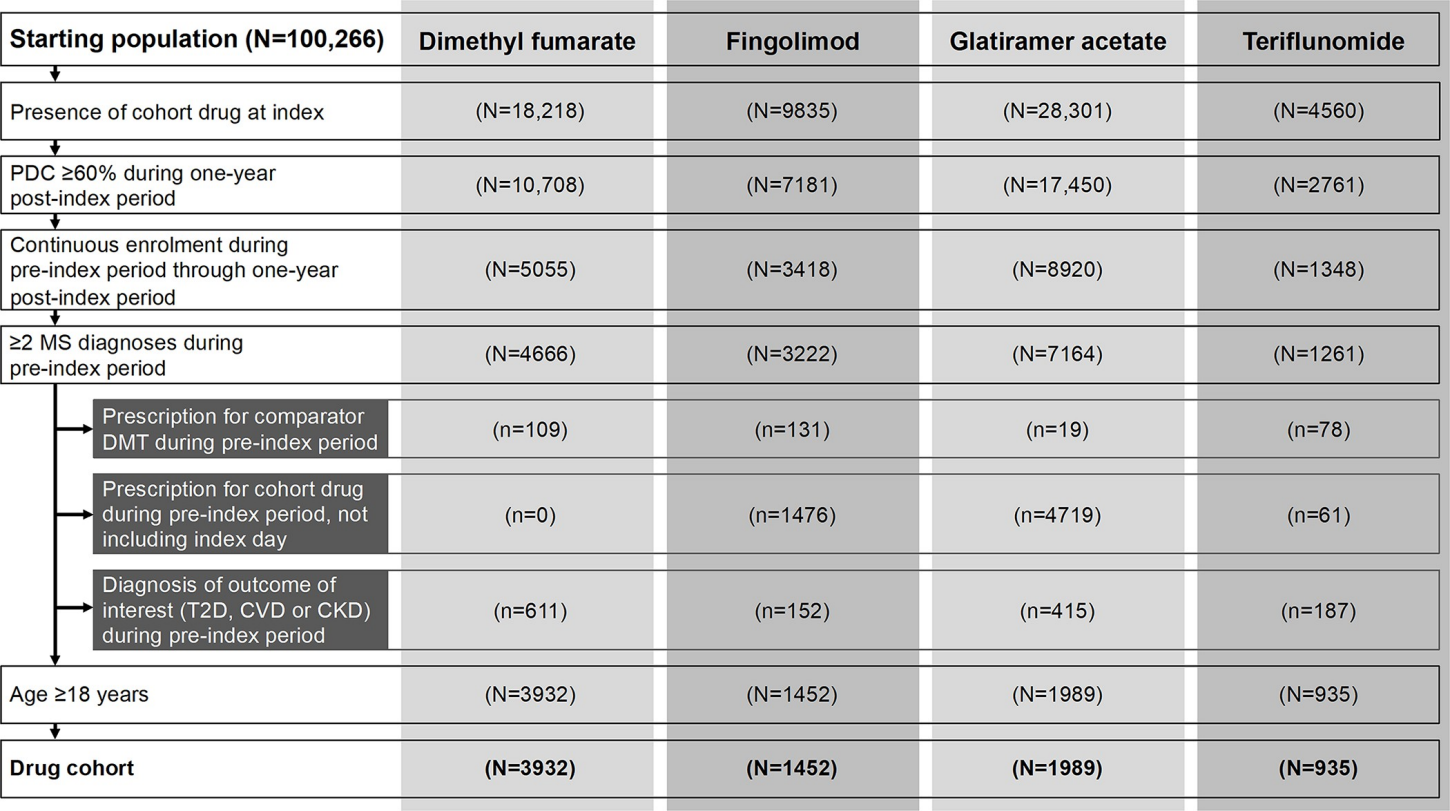
Attrition diagram for four disease-modifying therapy cohorts generated using inclusion/exclusion criteria. CKD, chronic kidney disease; CVD, cardiovascular disease; DMT, disease modifying therapy; PDC, proportion of days covered; MS, multiple sclerosis; T2D, type 2 diabetes.

An attrition diagram illustrating the generation of the four DMT cohorts after application of all inclusion and exclusion criteria is presented in Fig 2. The dimethyl fumarate, fingolimod, glatiramer acetate and teriflunomide cohorts included individual-level data on 3932, 1452, 1989 and 935 patients, respectively.

### Descriptive analyses

There were several statistically significant differences between the four DMT cohorts at baseline, as presented in Table 1. Demographic differences included the mean age at index, the proportion of female patients, geographical distribution of patients and payer type. Regarding clinical risk factors, tobacco use was more common among glatiramer acetate and teriflunomide patients than among dimethyl fumarate patients. The use of mobility aids, a proxy for MS severity, was more common among patients receiving dimethyl fumarate than patients receiving fingolimod and glatiramer acetate. Both hyperlipidaemia and hypertension were more common in the teriflunomide cohort than in the dimethyl fumarate cohort, but less common in the fingolimod and glatiramer acetate cohorts than in the dimethyl fumarate cohort. This was only statistically significant when the dimethyl fumarate cohort was compared to the fingolimod cohort. The Charlson score was higher on average in the glatiramer acetate and teriflunomide groups than in the dimethyl fumarate group, but lower in the fingolimod group than in the dimethyl fumarate group.

**Table 1:** Patient characteristics by disease-modifying therapy cohort.

Baseline characteristic	Dimethyl fumarate cohort (N = 3932)	Fingolimod cohort (N = 1452)	Glatiramer acetate cohort (N = 1989)	Teriflunomide cohort (N = 935)	*p*-value dimethyl fumarate vs fingolimod	*p*-value dimethyl fumarate vs glatiramer acetate	*p*-value dimethyl fumarate vs teriflunomide
Mean (SD) age at index, years	46.0 (10.7)	42.9 (10.5)	43.1 (11.2)	50.1 (9.9)	<0.001*^†^	<0.001*^†^	<0.001*^†^
Female	2924 (74.4)	1094 (75.3)	1536 (77.2)	734 (78.5)	0.480^‡^	0.017^‡^	0.009^‡^
Region					<0.001^§^	0.392^§^	<0.001^§^
Midwest	885 (22.5)	361 (24.9)	428 (21.5)	238 (25.5)			
Northeast	894 (22.7)	275 (18.9)	429 (21.6)	169 (18.1)			
South	1468 (37.3)	622 (42.8)	786 (39.5)	391 (41.8)			
West	685 (17.4)	194 (13.4)	346 (17.4)	137 (14.7)			
Tobacco use (yes)	235 (6.0)	108 (7.4)	184 (9.3)	86 (9.2)	0.059^‡^	<0.001^‡^	<0.001^‡^
Payer type					0.001^‡^	0.018^‡^	<0.001^‡^
Commercial	3792 (96.4)	1425 (98.1)	1941 (97.6)	875 (93.6)			
Medicare	140 (3.6)	27 (1.9)	48 (2.4)	60 (6.4)			
Use of mobility aids^¶^ (yes)	168 (4.3)	44 (3.0)	64 (3.2)	48 (5.1)	0.040^‡^	0.055^‡^	0.251^‡^
Hyperlipidaemia (yes)	927 (23.6)	243 (16.7)	403 (20.3)	299 (32.0)	<0.001^‡^	0.004^‡^	<0.001^‡^
Hypertension (yes)	1227 (31.2)	343 (23.6)	521 (26.2)	350 (37.4)	<0.001^‡^	<0.001^‡^	<0.001^‡^
Mean (SD) Charlson score	0.35 (0.8)	0.29 (0.7)	0.47 (1.0)	0.45 (1.0)	0.007*	<0.001*	0.001*
**Follow-up variable**							
Duration of follow-up, days					<0.001*^†^	<0.001*^†^	<0.001*^†^
Mean (SD)	1268.7 (654.0)	1145.8 (594.2)	1149.2 (599.1)	1060.3 (576.1)			
Median (Q1, Q3)	1161 (655.0, 1868.5)	1017 (626.5, 1614.0)	1035 (631.0, 1581.0)	919 (596.0, 1395.0)			
Discontinued index drug (yes)	1850 (47.0)	571 (39.3)	1101 (55.4)	594 (63.5)	<0.001^‡^	<0.001^‡^	<0.001^‡^
Time to discontinuation of index drug, days^**‖**^					0.060*	<0.001*^†^	0.012*
					0.169^†^		0.396^†^
Mean (SD)	652.6 (434.9)	613.6 (422.6)	553.1 (380.9)	706.4 (514.4)			
Median (Q1, Q3)	516 (337.0, 859.0)	479 (322.0, 782.0)	425 (308.0, 682.0)	535 (344.0, 957.0)			

All results are presented as n (%) unless stated otherwise.

The only missing data imputed were Region. Missing data were imputed to mode.

*p-value from ANOVA

^†^*p*-value from Mood’s median test

^‡^*p*-value from Fisher’s exact test

^§^*p*-value from Monte Carlo Fisher’s exact test.

^¶^Use of mobility aids was included as a proxy for disease severity.

^**‖**^For those patients who discontinued index drug only (N for each DMT cohort in row above).

DMT, dimethyl fumarate; Q1, first quartile; Q3, third quartile; SD, standard deviation.

With respect to follow-up variables, the median duration of follow-up was statistically significantly higher in the dimethyl fumarate cohort than in the other three DMT cohorts (Table 1). The teriflunomide cohort had the largest proportion of patients who discontinued their index drug, followed by the glatiramer acetate cohort, while the fingolimod cohort had the smallest proportion of patients who discontinued. The glatiramer acetate cohort had the shortest median time to discontinuation of index drug, which was significantly lower than in the dimethyl fumarate cohort; however, median time to discontinuation was similar in the dimethyl fumarate and the other two DMT cohorts.

**Fig 3:**
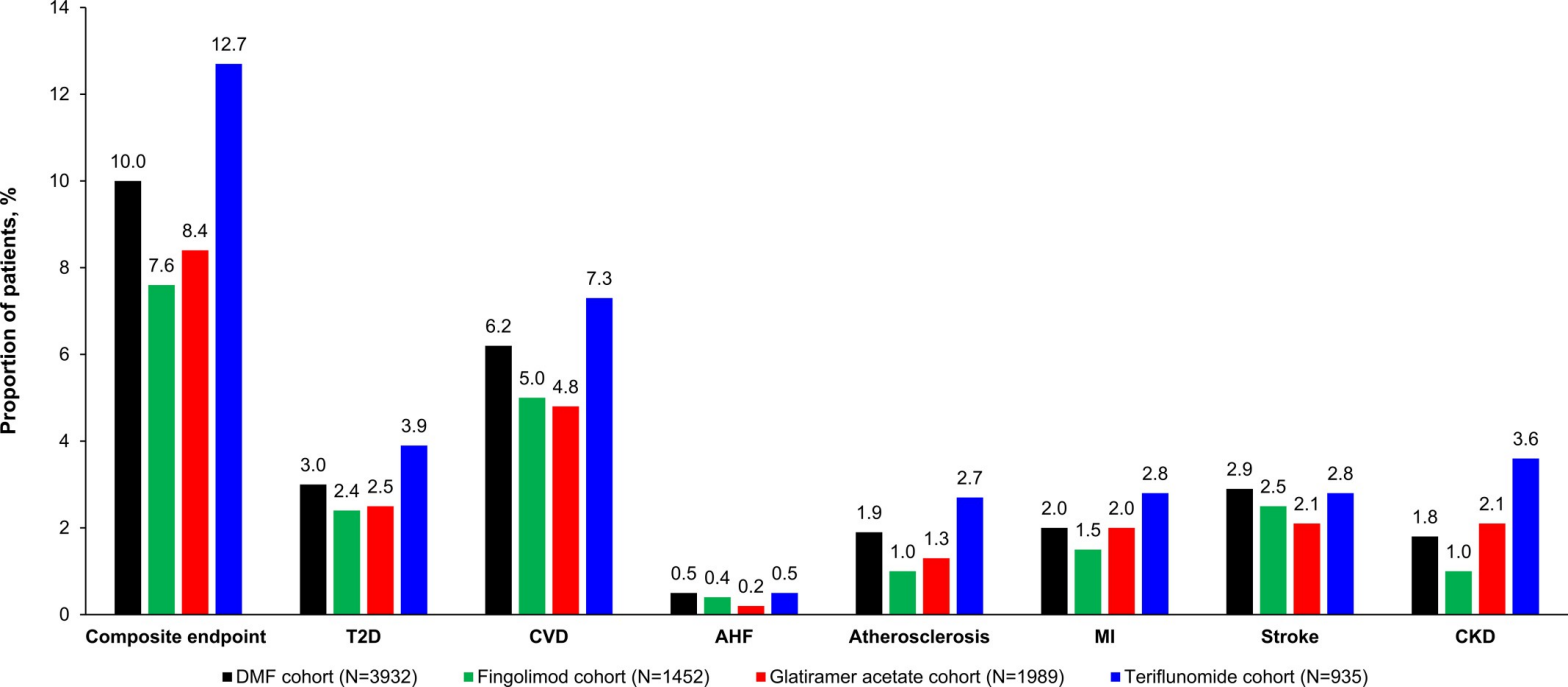
Descriptive analyses: Unadjusted incidence of composite endpoint and individual outcomes by disease-modifying therapy cohort. AHF, acute heart failure; CKD, chronic kidney disease; CVD, cardiovascular disease; DMF, dimethyl fumarate; MI, myocardial infarction; T2D, type 2 diabetes.

The observed proportions of patients with the composite endpoint and the individual outcomes for each of the four cohorts are presented in Fig 3. The teriflunomide cohort had the largest proportion of patients with composite endpoint incidence, followed by the dimethyl fumarate cohort. The fingolimod patient cohort had the smallest proportion of patients with incidence of the composite endpoint. A similar pattern was observed for each of the individual outcomes.

**Fig 4:**
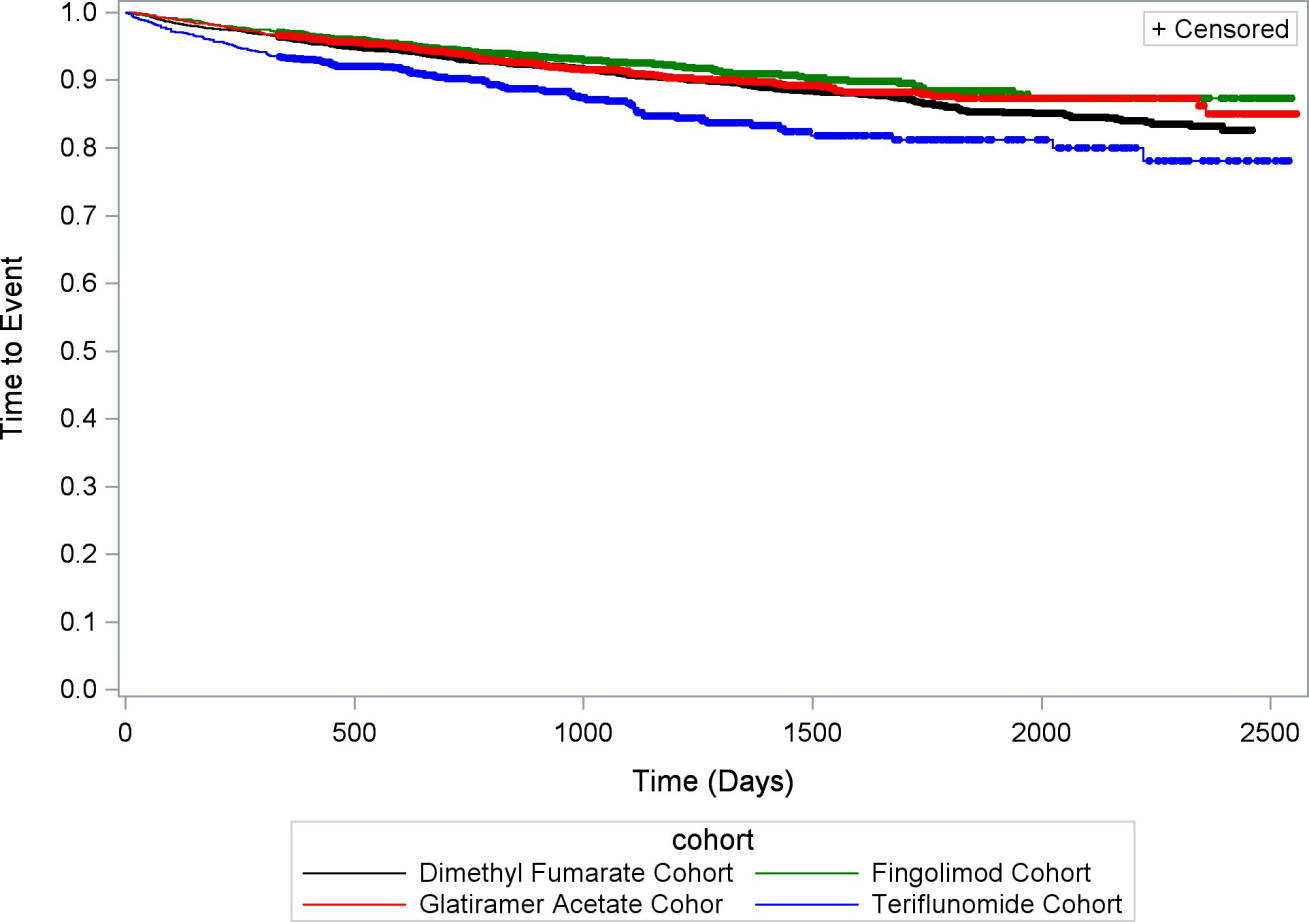
Unadjusted Kaplan–Meier estimates of time to first occurrence of composite endpoint. Results for the dimethyl fumarate, fingolimod, glatiramer acetate and teriflunomide cohorts.

The time to the first occurrence of the composite endpoint for the four different cohorts is shown in the unadjusted Kaplan–Meier curve in Fig 4. All treatments showed similar profiles, although the teriflunomide patient cohort had an earlier occurrence of the composite endpoint.

### Comparative analyses

The forest plot in Fig 1 shows all the analysis strategies implemented in the data-driven comparative analysis approach comparing dimethyl fumarate and teriflunomide cohorts for the composite endpoint TTE analysis. Both the FMA and the best model, selected by minimum cross-validated mean square prediction error (CvMSPE), are also displayed. In addition, each of the analysis strategies highlights the consistency and robustness of the approaches used in estimating the treatment effect.

**Fig 5:**
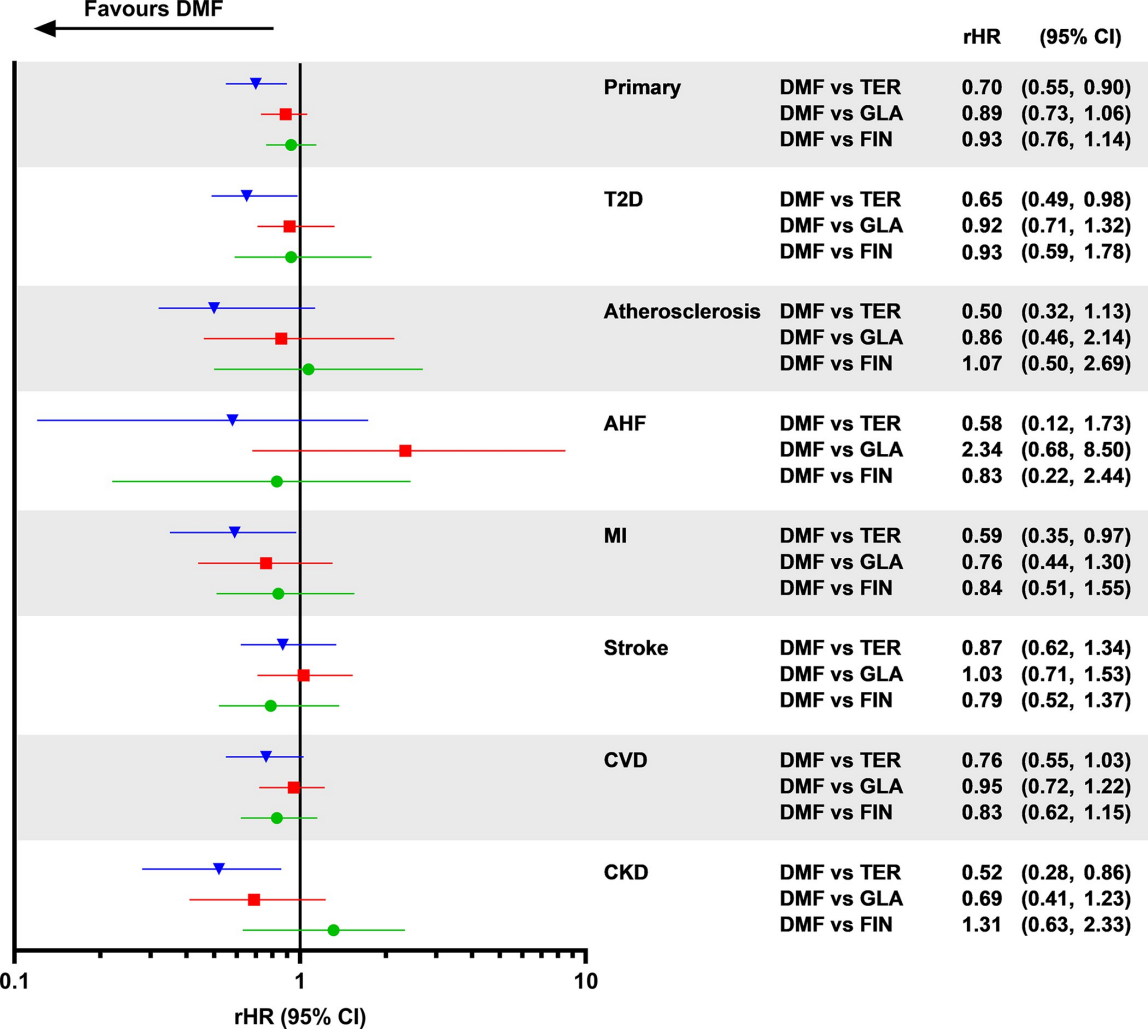
Forest plot for composite endpoint and individual outcome time to event analyses. This is based on the frequentist model average result for dimethyl fumarate versus teriflunomide, dimethyl fumarate versus glatiramer acetate and dimethyl fumarate versus fingolimod. AHF, acute heart failure; CKD, chronic kidney disease; CVD, cardiovascular disease; DMF, dimethyl fumarate; FIN, fingolimod; GLA, glatiramer acetate; MI, myocardial infarction; rHR, restricted hazard ratio; T2D, type 2 diabetes; TER, teriflunomide.

A forest plot for the composite endpoint and individual outcome TTE analyses, based on the FMA results for dimethyl fumarate versus fingolimod, dimethyl fumarate versus glatiramer acetate and dimethyl fumarate versus teriflunomide, is shown in Fig 5. For the composite endpoint, there was a statistically significantly lower risk of incidence for dimethyl fumarate versus teriflunomide (rHR [95% CI] 0.70 [0.55, 0.90]) but no difference for the other two DMTs. Regarding individual outcomes, there was a statistically significantly lower risk of T2D (rHR [95% CI] 0.65 [0.49, 0.98]), MI (0.59 [0.35, 0.97]) and CKD (0.52 [0.28, 0.86]) incidence for dimethyl fumarate versus teriflunomide, but no difference for other individual outcomes or for dimethyl fumarate versus glatiramer acetate or fingolimod.

### Sensitivity analyses

The descriptive and comparative results of the sensitivity analyses in only those patients who discontinued with their index drug are presented in the **Supporting information**. S2 Fig shows the sensitivity analysis unadjusted Kaplan–Meier curve for time to the first occurrence of the composite endpoint for the four cohorts. Like the main analysis, all treatments showed similar profiles, although the teriflunomide patient cohort showed an earlier occurrence of the composite endpoint. A forest plot for the composite endpoint and the individual outcome TTE sensitivity analyses based on the FMA results is shown in S3 Fig. For the composite endpoint, there was a statistically significantly lower risk of incidence for dimethyl fumarate versus teriflunomide (rHR [95% CI] 0.68 [0.53, 0.90]) and glatiramer acetate (0.80 [0.65, 0.99]), but no statistical difference for dimethyl fumarate versus fingolimod. For the individual outcomes, there was a statistically significantly lower risk of T2D (rHR [95% CI] 0.63 [0.46, 0.94]), MI (0.55 [0.29, 0.90]), CVD (0.73 [0.53, 0.996]) and CKD (0.46 [0.25, 0.81]) incidence for dimethyl fumarate versus teriflunomide, but no difference for other individual outcomes or for dimethyl fumarate versus glatiramer acetate or fingolimod.

## Discussion

The aim of this study was to determine whether dimethyl fumarate, through its effect on the Nrf2 pathway, might result in a decreased incidence of T2D, CVD and CKD, in patients with MS, using real-world data derived from US administrative claims databases. In this way, we hoped to provide human evidence (i.e., target validation) in support of drug discovery efforts targeting the Nrf2 pathway for these indications prior to their use in the clinic. Standardised electronic healthcare databases are commonly used at multiple stages in drug development. While they have proven valuable, causal relationships are notoriously hard to establish in studies without randomisation. Simple self-controlled cohort studies have the potential for high false-positive discovery rates due to multiple drug-outcome comparisons and for the underestimation of within-subject variability [1]; more sophisticated approaches are required to reliably investigate comparative effectiveness using claims databases.

Traditional methods of balancing groups in a non-randomised setting, such as regression, stratification, matching and weighting, aim to adjust for selection bias and confounding [13]. Propensity score methods have become a very popular approach to achieving comparability of treatment groups on pre-treatment covariates, although their ability to do so depends on the extent to which measured variables capture any potential confounding [24]. The appropriate application of propensity score matching can select real-world subgroups of individuals whose demographics and baseline characteristics are as balanced as if they had been randomised in a clinical trial. However, using unadjusted or incorrectly adjusted models can lead to misleading or incorrect results [25].

In a typical analysis, and with the plethora of possibilities in selecting an ‘appropriate’ model strategy, it is difficult to be certain that the correct model will be chosen, and that the reported results will be accurate. FMA is an innovative data-driven approach that utilises model averaging to decrease the chance of model misspecification and may be particularly useful in scenarios with complex confounding [13]. Therefore, the results of the present analysis are more robust than traditional approaches, which usually specify only one or, at best, two model strategies. Nevertheless, repeating this analysis on multiple data sets would add an additional layer of robustness [26]. Using FMA, we found that patients treated with dimethyl fumarate trended toward a decreased incidence of T2D, CVD and CKD relative to other DMTs. However, this study did not provide definitive statistical support for the hypothesis that Nrf2 activation would provide additional metabolic disease benefit in patients with MS.

With more advanced methods in the causal inference space, administrative claims data are becoming an increasingly reliable source of evidence to use for the purpose of bridging the translational gap between animals and humans, where drugs in development often fail. Furthermore, by using existing real-world data on a large scale, drug development can be advanced in a shorter timeframe without the need for expensive clinical trials that may place a burden and risk on participants. While this approach cannot fully replace the gold standard of randomised clinical trials, it may help reduce the number of early phase trials of drugs that ultimately fail to reach approval. In addition, licenced drugs often have well-described safety profiles, reducing the risk of unexpected adverse events when applied to other indications. The novel FMA approach used in this study could serve as a model for future non-randomised comparative analyses using administrative claims data–ultimately contributing to a more efficient drug discovery process.

Limitations of this study include possible influence from unmeasured confounders due to the observational study design. Administrative claims data are collected for reimbursement purposes, not for assessing treatment effectiveness, and are subject to coding errors or omissions. Baseline data such as tobacco use quantified in pack years and clinical measures such as MS severity, rate of progression, MS-related disability (such as Expanded Disability Status Scale [EDSS]), obesity and laboratory test results (e.g., glycated haemoglobin, blood pressure, renal retention parameters) are not available in claims data; these may impact treatment decisions and could not be controlled for. Mobility aids were used as a proxy for disease severity but certainly do not fully reflect the clinical disease severity of an individual. It was not possible to differentiate between patients using a unilateral or bilateral walking aid/wheelchair; however, use of mobility aids was relatively uncommon in each cohort. The comparative analyses did not adjust for multiple testing. The limited follow-up period in this study may not be sufficient to capture the long-term effects of the treatments or detect delayed or cumulative adverse events. Finally, Merative L.P.^®^ Research Databases contain data from patients with employer-sponsored health plans or Medicare supplemental insurance and may not be representative of the US as a whole or comparable to other countries and healthcare systems.

## Conclusions

This study effectively demonstrated the use of an innovative statistical FMA framework based on machine learning to test a clinical hypothesis using existing non-randomised real-world data on a large scale to perform early target validation for drug discovery. Our results suggest that patients with MS treated with dimethyl fumarate, a Nrf2 pathway agonist, may have an advantage over those receiving teriflunomide with respect to the occurrence of T2D and its complications. This was evidenced by statistically significant differences in the rHRs between the DMF and teriflunomide treatment groups in both the main and sensitivity analyses supporting the differences in incidence described in the data. For the other treatment groups, there was a trend among patients treated with DMF towards a decreased incidence of T2D, CVD and CKD relative to other disease-modifying therapies, although this was not statistically significant. This retrospective study using real-world data was intended to affirm a mechanistic hypothesis, and not to yield a definitive conclusion as to whether Nrf2 activation provides additional metabolic disease benefit in patients with MS. However, the data generated in this study are useful in that they provide real-world data in humans to support prospective clinical trials to optimally test this hypothesis.

## Supporting information

S1 Fig Study time periods. DMT, disease-modifying therapy; MS, multiple sclerosis; PDC, proportion of days covered.
(TIF)

S2 Fig Sensitivity analysis: Unadjusted Kaplan–Meier estimates of time to first occurrence of composite endpoint. Results for the dimethyl fumarate, fingolimod, glatiramer acetate and teriflunomide cohorts.
(TIF)

S3 Fig Forest plot for composite endpoint and individual outcome sensitivity time to event analyses. This is based on the frequentist model average result for dimethyl fumarate versus teriflunomide, dimethyl fumarate versus glatiramer acetate and dimethyl fumarate versus fingolimod. AHF, acute heart failure; CKD, chronic kidney disease; CI, confidence interval; CVD, cardiovascular disease; DMF, dimethyl fumarate; FIN, fingolimod; GLA, glatiramer acetate; MI, myocardial infarction; rHR, restricted hazard ratio; T2D, type 2 diabetes; TER, teriflunomide.
(TIF)

S1 Table Codes for incident variables used in the primary composite endpoint. (PDF)
