# Supplementary material for: Incidence of type 2 diabetes, cardiovascular disease and chronic kidney disease in patients with multiple sclerosis initiating disease-modifying therapies: Retrospective cohort study using a frequentist model averaging statistical framework
Source: PLoS One. 2024 Mar 22;19(3):e0300708. doi: 10.1371/journal.pone.0300708 (PMC10959335; doi:10.1371/journal.pone.0300708)
Supplement: S1 Table — (PDF) [file pone.0300708.s004.pdf]

Type 2 diabetes mellitus codes [ICD-10]

Code	Description
E11.00	Type 2 diabetes mellitus with hyperosmolarity without nonketotic hyperglycemic-hyperosmolar coma (NKHHC)
E11.01	Type 2 diabetes mellitus with hyperosmolarity with coma
E11.10	Type 2 diabetes mellitus with ketoacidosis without coma
E11.11	Type 2 diabetes mellitus with ketoacidosis with coma
E11.21	Type 2 diabetes mellitus with diabetic nephropathy
E11.22	Type 2 diabetes mellitus with diabetic chronic kidney disease
E11.29	Type 2 diabetes mellitus with other diabetic kidney complication
E11.311	Type 2 diabetes mellitus with unspecified diabetic retinopathy with macular edema
E11.319	Type 2 diabetes mellitus with unspecified diabetic retinopathy without macular edema
E11.3211	Type 2 diabetes mellitus with mild nonproliferative diabetic retinopathy with macular edema, right eye
E11.3212	Type 2 diabetes mellitus with mild nonproliferative diabetic retinopathy with macular edema, left eye
E11.3213	Type 2 diabetes mellitus with mild nonproliferative diabetic retinopathy with macular edema, bilateral
E11.3219	Type 2 diabetes mellitus with mild nonproliferative diabetic retinopathy with macular edema, unspecified eye
E11.3291	Type 2 diabetes mellitus with mild nonproliferative diabetic retinopathy without macular edema, right eye
E11.3292	Type 2 diabetes mellitus with mild nonproliferative diabetic retinopathy without macular edema, left eye
E11.3293	Type 2 diabetes mellitus with mild nonproliferative diabetic retinopathy without macular edema, bilateral
E11.3299	Type 2 diabetes mellitus with mild nonproliferative diabetic retinopathy without macular edema, unspecified eye
E11.3311	Type 2 diabetes mellitus with moderate nonproliferative diabetic retinopathy with macular edema, right eye
E11.3312	Type 2 diabetes mellitus with moderate nonproliferative diabetic retinopathy with macular edema, left eye
E11.3313	Type 2 diabetes mellitus with moderate nonproliferative diabetic retinopathy with macular edema, bilateral
E11.3319	Type 2 diabetes mellitus with moderate nonproliferative diabetic retinopathy with macular edema, unspecified eye
E11.3391	Type 2 diabetes mellitus with moderate nonproliferative diabetic retinopathy without macular edema, right eye
E11.3392	Type 2 diabetes mellitus with moderate nonproliferative diabetic retinopathy without macular edema, left eye
E11.3393	Type 2 diabetes mellitus with moderate nonproliferative diabetic retinopathy without macular edema, bilateral
E11.3399	Type 2 diabetes mellitus with moderate nonproliferative diabetic retinopathy without macular edema, unspecified eye
E11.3411	Type 2 diabetes mellitus with severe nonproliferative diabetic retinopathy with macular edema, right eye
E11.3412	Type 2 diabetes mellitus with severe nonproliferative diabetic retinopathy with macular edema, left eye
E11.3413	Type 2 diabetes mellitus with severe nonproliferative diabetic retinopathy with macular edema, bilateral
E11.3419	Type 2 diabetes mellitus with severe nonproliferative diabetic retinopathy with macular edema, unspecified eye
E11.3491	Type 2 diabetes mellitus with severe nonproliferative diabetic retinopathy without macular edema, right eye
E11.3492	Type 2 diabetes mellitus with severe nonproliferative diabetic retinopathy without macular edema, left eye
E11.3493	Type 2 diabetes mellitus with severe nonproliferative diabetic retinopathy without macular edema, bilateral
E11.3499	Type 2 diabetes mellitus with severe nonproliferative diabetic retinopathy without macular edema, unspecified eye
E11.3511	Type 2 diabetes mellitus with proliferative diabetic retinopathy with macular edema, right eye
E11.3512	Type 2 diabetes mellitus with proliferative diabetic retinopathy with macular edema, left eye
E11.3513	Type 2 diabetes mellitus with proliferative diabetic retinopathy with macular edema, bilateral
E11.3519	Type 2 diabetes mellitus with proliferative diabetic retinopathy with macular edema, unspecified eye
E11.3521	Type 2 diabetes mellitus with proliferative diabetic retinopathy with traction retinal detachment involving the macula, right eye
E11.3522	Type 2 diabetes mellitus with proliferative diabetic retinopathy with traction retinal detachment involving the macula, left eye
E11.3523	Type 2 diabetes mellitus with proliferative diabetic retinopathy with traction retinal detachment involving the macula, bilateral
E11.3529	Type 2 diabetes mellitus with proliferative diabetic retinopathy with traction retinal detachment involving the macula, unspecified eye
E11.3531	Type 2 diabetes mellitus with proliferative diabetic retinopathy with traction retinal detachment not involving the macula, right eye
E11.3532	Type 2 diabetes mellitus with proliferative diabetic retinopathy with traction retinal detachment not involving the macula, left eye
E11.3533	Type 2 diabetes mellitus with proliferative diabetic retinopathy with traction retinal detachment not involving the macula, bilateral
E11.3539	Type 2 diabetes mellitus with proliferative diabetic retinopathy with traction retinal detachment not involving the macula, unspecified eye
E11.3541	Type 2 diabetes mellitus with proliferative diabetic retinopathy with combined traction retinal detachment and rhegmatogenous retinal detachment, right eye
E11.3542	Type 2 diabetes mellitus with proliferative diabetic retinopathy with combined traction retinal detachment and rhegmatogenous retinal detachment, left eye
E11.3543	Type 2 diabetes mellitus with proliferative diabetic retinopathy with combined traction retinal detachment and rhegmatogenous retinal detachment, bilateral
E11.3549	Type 2 diabetes mellitus with proliferative diabetic retinopathy with combined traction retinal detachment and rhegmatogenous retinal detachment, unspecified eye
E11.3551	Type 2 diabetes mellitus with stable proliferative diabetic retinopathy, right eye
E11.3552	Type 2 diabetes mellitus with stable proliferative diabetic retinopathy, left eye
E11.3553	Type 2 diabetes mellitus with stable proliferative diabetic retinopathy, bilateral
E11.3559	Type 2 diabetes mellitus with stable proliferative diabetic retinopathy, unspecified eye
E11.3591	Type 2 diabetes mellitus with proliferative diabetic retinopathy without macular edema, right eye
E11.3592	Type 2 diabetes mellitus with proliferative diabetic retinopathy without macular edema, left eye
E11.3593	Type 2 diabetes mellitus with proliferative diabetic retinopathy without macular edema, bilateral
E11.3599	Type 2 diabetes mellitus with proliferative diabetic retinopathy without macular edema, unspecified eye
E11.36	Type 2 diabetes mellitus with diabetic cataract
E11.37X1	Type 2 diabetes mellitus with diabetic macular edema, resolved following treatment, right eye
E11.37X2	Type 2 diabetes mellitus with diabetic macular edema, resolved following treatment, left eye
E11.37X3	Type 2 diabetes mellitus with diabetic macular edema, resolved following treatment, bilateral
E11.37X9	Type 2 diabetes mellitus with diabetic macular edema, resolved following treatment, unspecified eye
E11.39	Type 2 diabetes mellitus with other diabetic ophthalmic complication
E11.40	Type 2 diabetes mellitus with diabetic neuropathy, unspecified
E11.41	Type 2 diabetes mellitus with diabetic mononeuropathy
E11.42	Type 2 diabetes mellitus with diabetic polyneuropathy

E11.43	Type 2 diabetes mellitus with diabetic autonomic (poly)neuropathy
E11.44	Type 2 diabetes mellitus with diabetic amyotrophy
E11.49	Type 2 diabetes mellitus with other diabetic neurological complication
E11.51	Type 2 diabetes mellitus with diabetic peripheral angiopathy without gangrene
E11.52	Type 2 diabetes mellitus with diabetic peripheral angiopathy with gangrene
E11.59	Type 2 diabetes mellitus with other circulatory complications
E11.610	Type 2 diabetes mellitus with diabetic neuropathic arthropathy
E11.618	Type 2 diabetes mellitus with other diabetic arthropathy
E11.620	Type 2 diabetes mellitus with diabetic dermatitis
E11.621	Type 2 diabetes mellitus with foot ulcer
E11.622	Type 2 diabetes mellitus with other skin ulcer
E11.628	Type 2 diabetes mellitus with other skin complications
E11.630	Type 2 diabetes mellitus with periodontal disease
E11.638	Type 2 diabetes mellitus with other oral complications
E11.641	Type 2 diabetes mellitus with hypoglycemia with coma
E11.649	Type 2 diabetes mellitus with hypoglycemia without coma
E11.65	Type 2 diabetes mellitus with hyperglycemia
E11.69	Type 2 diabetes mellitus with other specified complication
E11.8	Type 2 diabetes mellitus with unspecified complications
E11.9	Type 2 diabetes mellitus without complications

Type 2 diabetes mellitus codes [ICD-9]

Code	Description
250.00	Diabetes mellitus without mention of complication, type II or unspecified type, not stated as uncontrolled
250.02	Diabetes mellitus without mention of complication, type II or unspecified type, uncontrolled
250.10	Diabetes with ketoacidosis, type II or unspecified type, not stated as uncontrolled
250.12	Diabetes with ketoacidosis, type II or unspecified type, uncontrolled
250.20	Diabetes with hyperosmolarity, type II or unspecified type, not stated as uncontrolled
250.22	Diabetes with hyperosmolarity, type II or unspecified type, uncontrolled
250.30	Diabetes with other coma, type II or unspecified type, not stated as uncontrolled
250.32	Diabetes with other coma, type II or unspecified type, uncontrolled
250.40	Diabetes with renal manifestations, type II or unspecified type, not stated as uncontrolled
250.42	Diabetes with renal manifestations, type II or unspecified type, uncontrolled
250.50	Diabetes with ophthalmic manifestations, type II or unspecified type, not stated as uncontrolled
250.52	Diabetes with ophthalmic manifestations, type II or unspecified type, uncontrolled
250.60	Diabetes with neurological manifestations, type II or unspecified type, not stated as uncontrolled
250.62	Diabetes with neurological manifestations, type II or unspecified type, uncontrolled
250.70	Diabetes with peripheral circulatory disorders, type II or unspecified type, not stated as uncontrolled
250.72	Diabetes with peripheral circulatory disorders, type II or unspecified type, uncontrolled
250.80	Diabetes with other specified manifestations, type II or unspecified type, not stated as uncontrolled
250.82	Diabetes with other specified manifestations, type II or unspecified type, uncontrolled
250.90	Diabetes with unspecified complication, type II or unspecified type, not stated as uncontrolled
250.92	Diabetes with unspecified complication, type II or unspecified type, uncontrolled

Diabetes medication codes [NDC]

Only the first 1000 codes have been included, to download the full codelist please contact Lilly

Code	Description
55175512502	
00364223250	
00364223350	
00591559703	
00591559800	
00591559803	
00603206021	
00603206121	
00719100610	
00719100710	
00839738604	
00839738704	
35470068501	
35470068601	
47202282901	
47202283001	
71610030009	
00003013950	
00003015250	
00093001005	
00102322505	
00157071910	
00157072305	
00172290260	
00172290265	
00172290270	
00172290280	
00172365760	
00172365770	
00172404070	
00172404161	
00172404165	
00182115901	
00182115905	
00182185105	
00182185217	
00302107005	
00302107010	
00302107505	
00302107510	

00302107525	
00304135100	
00304135101	
00304135105	
00304135200	
00304135201	
00306299090	
00306299185	
00339506412	
00339506512	
00364069905	Allopurinol
00367215310	
00367215350	
00367215396	
00367215510	
00367215550	
00403005201	
00403005230	
00403005401	
00403005430	
00403005471	
00454007701	
00454007801	
00454007810	
00537632105	
00555035004	
00556039205	
00556039210	
00615254029	
00615254129	
00693021001	
00693021005	
00693021010	
00693021705	
00719121910	
00719121912	
00779023627	
00814169528	
00832096925	
00839627712	
00839627716	
00839627720	
11289154204	
11845021101	

11845021104
11845021201
11845021204
12071042201
12071042210
12071042501
12071042510
17236021010
17236021210
24208005101
24208005110
24208005111
24208005201
24208005204
24208005205
24208005210
24208005211
38022035801
38022035901
45124012401
45124012405
45124012410
47202272501
47202272503
47202273001
49334814902
49334814905
49334814906
49648135100
49648135101
49648135105
49648135200
49648135201
50272097003
51728073501
51728073505
51728073510
51728073601
51728073605
52446011428
52493052230
52493052301
52555025101
52555025110

52555025201
52728002595
52728014209
52765107600
53445185200
53467016701
53467016730
53467016801
53489021005
53633007710
53633007711
53633007801
53633007811
54124027630
54274000610
54274000650
54274000710
54274000730
54274000750
54274011850
54274026250
54274061610
54274061650
54274061810
54274061850
54569020200
54569020201
54569020202
54569020217
54569020232
54569020302
54569020303
54569020304
54569020317
54569020350
54569161400
54569201700
54569502900
54807012201
54807012301
54812019001
54812019010
54812019101
54812019110

54868088001	
55081013200	
55081013300	
55081013301	
55084051001	
55084051101	
55084051130	
55084051160	
55153103601	
55175385700	
55175385705	
55175385801	
55175385803	
55496150109	
55496150209	
57362028711	
57362028713	
57362028784	
57480031606	
58469346230	
58469346530	
60346063330	
60346063360	
60346063390	
60346078430	
00310677030	Qtern
71610017715	
71610017730	
71610017745	
23629002201	Fosinopril Sodium
43063069830	Glimepiride
43063094693	Glimepiride
50090317201	
50090317202	
50090317203	
50090317204	
50090318901	
50090318903	
50090318904	
50268035811	
50268035815	Glimepiride
50268035911	
50268035915	Glimepiride
50268036011	

50268036015	Glimepiride
58016046700	Glimepiride
61919025004	
61919025030	
61919025082	
61919044830	
61919044890	
61919072330	
70934016790	
70934017390	
70934032390	
71610023660	
71610023680	
00615359529	
00615359543	
00615359565	
00615359629	
00615359643	
00615359665	
42291042910	GlipiZIDE
42291043010	GlipiZIDE
43353037945	
50090306902	
51079081023	
54124069530	
54124073530	
54569020600	
54569020700	
54569020701	
54569020702	
55045205801	
55081025200	
55081061400	
55175385503	
55175385603	
55175517503	
55289002790	
55887096830	Famotidine
55953052401	
55953052470	
55953052480	
55953052501	
55953052570	
55953052580	

57362052084
57866023601
60346045730
60346061330
60760016530
61919028730
61919028782
61919071330
61919071390
61919071530
61919071590
61919085090
62584071633
62584071701
62584071733
62939541101
62939542101
63739011602
63739011702
68030646201
70934039190
71610017545
71610017573
71610017592
71610017594
71610017598
71205027090
00536564205
00536564310
00615155627
00615155643
00615450929
00615450943
00615450953
00615450963
51655075352
51655094724
51655094725
53506080330
53506082130
53506082230
53506083930
54124005230
54124005260

54124017130	
54124050130	
54569020001	
54569020002	
54569020003	
54569020004	
54569020005	
54569020400	
54569020401	
54569020402	
54569021000	
54569021001	
54569021100	
54569021101	
54569021102	
54569021103	
54569383103	
54569509800	
54868170701	
55175523001	
57480040801	
57480040806	
57480040901	
57480040906	
57866023701	
60346066230	
60346073030	
60346073060	
60346089030	
60346093830	
60429008412	
60429008460	
60429008490	
61392012030	GlyBURIDE
61392012090	GlyBURIDE
62584071801	
62584071833	
62584071901	
62584071933	
63739011801	
63739011802	
63739011803	
63739011901	
63739011903	

65243017627	
65243034309	
65243034336	
67544009853	
67544009860	
67544009870	
67544009880	
67544009890	
67544009892	
67544009894	
75834020201	GlyBURIDE
75834020301	GlyBURIDE
75834020400	GlyBURIDE
75834020401	GlyBURIDE
52544056010	
00172571000	
00172571100	
00172571200	
54569521001	
66116023330	
68030799101	
32849050081	
32849050087	
00169320511	
00169266211	Tresiba
00088221901	
53002080172	
54569165102	
00003352115	
32849075601	
00003270210	
00003020001	
32849080601	
32849081601	
54569281600	
54569281601	
54569281701	
54569289100	
54569289101	
00002751501	
66733077301	Insulin Lispro
66733082201	
66733082259	Insulin Lispro KwikPen
00002879857	

00003033301	
32849070601	
32849070602	
32849070607	
54569383501	
59060023144	
32849070801	
32849070802	
32849070803	
32849070807	
59060023174	
00003022201	
00003354215	
00003011101	
32849071701	
00403344918	
32849070701	
32849070702	
32849070707	
47918089818	Afrezza 8 unit (90)- 12 unit (90)
54569231901	
54569383301	
00338012612	Myxredlin
32849072701	
00003352715	
32849075501	
32849070501	
54569255701	
54569383400	
54569383401	
00003183110	
00003270310	
00003244310	
32849080501	
32849075701	
00003010001	
00003270110	
32849080701	
32849081701	
54569165200	
54569165202	
00003030001	
00440756295	
00615458053	

11788003760	MetFORMIN (Eqv-Fortamet)
11788003860	MetFORMIN (Eqv-Fortamet)
16714093801	MetFORMIN (Eqv-Glumetza)
16714093901	MetFORMIN (Eqv-Glumetza)
17236013201	
17236013301	
23155010209	MetFORMIN Hydrochloride
23155010309	MetFORMIN Hydrochloride
23155010409	MetFORMIN Hydrochloride
42806040560	MetFORMIN (Eqv-Fortamet)
42806040660	MetFORMIN (Eqv-Fortamet)
43063089820	MetFORMIN Hydrochloride
43063090230	MetFORMIN Hydrochloride ER
43353098270	
50090306400	
50090306405	
50090306406	
50228010501	MetFORMIN Hydrochloride
50228010505	MetFORMIN Hydrochloride
50228010601	MetFORMIN Hydrochloride
50228010701	MetFORMIN Hydrochloride
50228010710	MetFORMIN Hydrochloride
51224002050	MetFORMIN Hydrochloride
51224002060	MetFORMIN Hydrochloride
51224002070	MetFORMIN Hydrochloride
51224012050	MetFORMIN Hydrochloride
51224012060	MetFORMIN Hydrochloride
51224012070	MetFORMIN Hydrochloride
51224022050	MetFORMIN Hydrochloride
51224022060	MetFORMIN Hydrochloride
51224022070	MetFORMIN Hydrochloride
54569420201	
61919031390	
61919039782	
61919039790	
61919099060	
61919099090	
66116028260	
66116029330	
66116045430	
66116069560	
68030905601	
68645058259	
68645058359	

68645058459	
70010049210	
70934031190	
70934039990	
70934040490	
70934042790	
71610002080	
71610002094	
71610027860	
71610027880	
71610030480	
71610030494	
72578003601	MetFORMIN Hydrochloride ER
72789000930	MetFORMIN (Eqv-Glucophage XR)
72789000960	MetFORMIN (Eqv-Glucophage XR)
72789000990	MetFORMIN (Eqv-Glucophage XR)
72789000993	MetFORMIN (Eqv-Glucophage XR)
68405801916	
68405801116	
54569521300	
75834020501	Nateglinide
75834020601	Nateglinide
57237021990	Pioglitazone Hydrochloride
57237022090	Pioglitazone Hydrochloride
60687039101	Pioglitazone Hydrochloride
60687039111	
61919078130	
54569522500	
57866006909	
57866126402	
57866136403	
65243019509	
65243019512	
00169430313	Rybelsus
00169430713	Rybelsus
00169431413	Rybelsus
00003019550	
00003027750	
00003027760	
00003027775	
00009047704	
00032472502	
00093051201	
00093051301	

00172298080
00302718210
00304134501
00304134600
00304134601
00304134602
00304134605
00304135301
00306663680
00306663780
00306663785
00306663880
00306663885
00349841700
00349841900
00349841910
00364072205
00367241210
00367241410
00367241421
00367241450
00367241496
00367241610
00367241650
00378021720
00405502502
00527107601
00527107610
00527119101
00527122601
00527122605
00536043801
00591558202
00615254401
00615254410
00615254413
00719197119
00781193205
00814792014
00814792114
00814792120
00814792130
00814792214
00832096710

00832096720
00832096750
00832096810
00832096825
00832096850
00832103613
00839701506
00839701516
00904023470
00904023508
11845036101
11845036104
11845036201
11845036203
17236032701
17236032705
17236032801
17236032805
17236032901
17236032905
24208018901
24208018911
24208019001
24208019005
24208019006
24208019010
24208019011
24208019101
24208019110
35470025801
35470025901
35470025909
35470026001
47202251001
47202274601
47202274603
47202274701
49648134501
49648134601
49648134602
49648134605
49648135301
49727094702
49727094802

49727094805
49727094902
49727094904
49884012210
49884012310
51079029140
51079029240
51079029340
51285032620
51728067001
51728067005
51728067010
51728067101
51728067201
51728067205
52446047532
52446047728
52555012301
52555012310
52555012401
52555022201
52555029210
52728009295
53002102000
53258017301
53258017313
53445164501
53467297901
53467297930
53489015105
53506055230
54274006610
54274006710
54274006750
54274006810
54274006830
54569020501
54569020502
54569020800
54569020801
54569020900
54569020901
54569167800
54807084001

54807084101
54807084201
55084057401
55084057501
55175385901
55175386001
58469473930
58469474430
00003069275
00009010010
00122047410
00150022340
00150022360
00150022380
00172224510
00187049201
00187049205
00188830201
00188830205
00188830210
00302720010
00304028001
00304028005
00306664380
00306664390
00339550912
00349217005
00349701210
00349873910
00359039310
00359039340
00359039350
00367244010
00367244050
00367244096
00536466807
00536466821
00537629101
00537629105
00537629110
00580035110
00615151432
00615151439
00686224510

00686224512
00686406213
00725206301
00725206310
00779113025
00779113027
00779113030
00781170413
00948528901
00948528910
10754098701
10876050501
10876050505
11289216802
11289216804
11289216805
12071035001
12071035005
17022871202
17022871204
17236029501
17236029505
17236029510
38022030401
45124075901
45124075905
45124075910
46198040701
46198040705
46703008705
47679030001
47679030065
49335122401
49335122402
49335122405
49727031704
51432046203
51728031301
51728031310
53258016201
53258016213
53445108400
53506055130
54124025102

54274057010	
54274057050	
54569021300	
54569021350	
54569021400	
54569021401	
54569021450	
54807051001	
54812028501	
54812028505	
55081471500	
55084052301	
55153112301	
57247103400	
57247103402	
57247103403	
57480038301	
57480038306	
58469466830	
54569469200	
54569469201	
54569469202	
54569469300	
54569469301	
54569469400	
00026286148	Precose
00026286151	Precose
00026286248	Precose
00026286251	Precose
00026286317	
00026286351	Precose
00054014020	
00054014025	Acarbose
00054014120	Acarbose
00054014125	Acarbose
00054014225	Acarbose
00115115001	Acarbose
00115115002	Acarbose
00115115003	
00115115101	Acarbose
00115115102	Acarbose
00115115103	
00115115201	Acarbose
00115115202	Acarbose

00115115203	
00179177870	
00179177871	
00179177872	
00378282010	Acarbose
00378282027	
00378282077	Acarbose
00378282110	Acarbose
00378282127	
00378282177	Acarbose
00378282210	Acarbose
00378282227	
00378282277	Acarbose
00855200350	
12527286104	
12527286109	
12527286209	
12527286309	
14288093625	
14288093720	
14288093725	
14288093825	
16252052300	
16252052301	Acarbose
16252052400	
16252052401	Acarbose
16252052500	
16252052501	Acarbose
23155014701	Acarbose
23155014705	
23155014710	
23155014801	Acarbose
23155014805	
23155014810	
23155014901	Acarbose
23155014905	
23155014910	
41284052301	
41284052400	
41284052401	
41284052500	
41284052501	
42291013010	
42291013090	Acarbose

42291013110	
42291013190	Acarbose
42291013210	
42291013290	Acarbose
43353090809	
43353095109	
47781034001	Acarbose
47781034101	Acarbose
47781034201	Acarbose
50419086148	Precose
50419086151	Precose
50419086251	Precose
50419086351	Precose
51129443001	
51129443002	
51862021001	Acarbose
51862021101	Acarbose
51862021201	Acarbose
53217013330	
53217013360	
53217013390	
54569450100	Precose
54569454800	Precose
54569606100	Acarbose
54868382300	
54868382301	Precose
54868583100	Precose
54868583101	Precose
54868594500	Acarbose
55289017142	Acarbose
63629160101	
63629160102	
63629569701	
63629569702	
63629569801	
63629569802	
63629569803	
64380075806	Acarbose
64380075906	Acarbose
64380076006	Acarbose
64896115001	
64896115003	
64896115101	
64896115103	

64896115201	
64896115203	
67263034601	
68151053701	
68788286103	
69543012010	Acarbose
69543012011	Acarbose
69543012110	Acarbose
69543012111	Acarbose
69543012210	Acarbose
69543012211	Acarbose
76439012010	Acarbose
76439012011	Acarbose
76439012110	Acarbose
76439012111	Acarbose
76439012210	Acarbose
76439012211	Acarbose
00002210303	
00002210322	Dymelor
00002210333	
00002210350	
00002210703	
00002210722	Dymelor
00002210733	
00002210750	Dymelor
00179025201	
00182185901	
00182186001	
00304184501	
00304184601	
00364223201	AcetoHEXAMIDE
00364223301	AcetoHEXAMIDE
00405402401	AcetoHEXAMIDE
00405402501	AcetoHEXAMIDE
00536300101	
00536300201	
00555044202	AcetoHEXAMIDE
00555044204	
00555044205	
00555044220	
00555044302	AcetoHEXAMIDE
00555044304	
00555044305	
00555044320	

00591559700	
00591559701	
00591559801	
00686044202	AcetoHEXAMIDE
00781169102	
00781169150	
00781169202	
00781169250	
00832101000	
00832101010	
00832101020	
00832101026	
00832101050	
00832101100	
00832101110	
00832101120	
00832101126	
00832101150	
00839738606	AcetoHEXAMIDE
00839738614	
00839738706	AcetoHEXAMIDE
00839738714	
00894593101	
00894593102	
00904199060	AcetoHEXAMIDE
00904199061	AcetoHEXAMIDE
00904199160	AcetoHEXAMIDE
00904199161	
17236045301	
51129157301	
51129536301	
51432002503	
51432003003	
51432003103	
54274023810	
54274023910	
00173086601	
00173086602	
00173086635	Tanzeum
00173086661	
00173086701	
00173086702	
00173086735	Tanzeum
00173086761	

45802008765	Alogliptin
45802010365	Alogliptin
45802015065	Alogliptin
64764012502	
64764012530	Nesina
64764012550	
64764012590	
64764025002	
64764025030	Nesina
64764025050	
64764025090	
64764062530	Nesina
64764062590	
45802023865	Alogliptin-Pioglitazone
45802026065	Alogliptin-Pioglitazone
45802030465	Alogliptin-Pioglitazone
45802035165	Alogliptin-Pioglitazone
45802040265	Alogliptin-Pioglitazone
45802049965	Alogliptin-Pioglitazone
64764012103	Oseni
64764012104	
64764012105	
64764012106	
64764012303	Oseni
64764012304	
64764012305	
64764012306	
64764012403	Oseni
64764012404	
64764012405	
64764012406	
64764025103	Oseni
64764025104	
64764025105	
64764025106	
64764025303	Oseni

Acute heart failure codes [ICD-10]

Code	Description
I50.21	Acute systolic (congestive) heart failure
I50.23	Acute on chronic systolic (congestive) heart failure
I50.31	Acute diastolic (congestive) heart failure
I50.33	Acute on chronic diastolic (congestive) heart failure
I50.41	Acute combined systolic (congestive) and diastolic (congestive) heart failure
I50.43	Acute on chronic combined systolic (congestive) and diastolic (congestive) heart failure

Acute heart failure codes [ICD-9]

Code	Description
428.21	Acute systolic heart failure
428.23	Acute on chronic systolic heart failure
428.31	Acute diastolic heart failure
428.33	Acute on chronic diastolic heart failure
428.41	Acute combined systolic and diastolic heart failure
428.43	Acute on chronic combined systolic and diastolic heart failure

Atherosclerosis codes [ICD-10]

Code	Description
I70.0	Atherosclerosis of aorta
I70.1	Atherosclerosis of renal artery
I70.209	Unspecified atherosclerosis of native arteries of extremities, unspecified extremity
I70.219	Atherosclerosis of native arteries of extremities with intermittent claudication, unspecified extremity
I70.229	Atherosclerosis of native arteries of extremities with rest pain, unspecified extremity
I70.399	Other atherosclerosis of unspecified type of bypass graft(s) of the extremities, unspecified extremity
I70.499	Other atherosclerosis of autologous vein bypass graft(s) of the extremities, unspecified extremity
I70.599	Other atherosclerosis of nonautologous biological bypass graft(s) of the extremities, unspecified extremity
I70.8	Atherosclerosis of other arteries
I70.90	Unspecified atherosclerosis
I70.91	Generalized atherosclerosis
I70.92	Chronic total occlusion of artery of the extremities

Atherosclerosis codes [ICD-9]

Code	Description
440.0	Atherosclerosis of aorta
440.1	Atherosclerosis of renal artery
440.20	Atherosclerosis of native arteries of the extremities, unspecified
440.21	Atherosclerosis of native arteries of the extremities with intermittent claudication
440.22	Atherosclerosis of native arteries of the extremities with rest pain
440.23	Atherosclerosis of native arteries of the extremities with ulceration
440.24	Atherosclerosis of native arteries of the extremities with gangrene
440.29	Other atherosclerosis of native arteries of the extremities
440.30	Atherosclerosis of unspecified bypass graft of the extremities
440.31	Atherosclerosis of autologous vein bypass graft of the extremities
440.32	Atherosclerosis of nonautologous biological bypass graft of the extremities
440.4	Chronic total occlusion of artery of the extremities
440.8	Atherosclerosis of other specified arteries
440.9	Generalized and unspecified atherosclerosis

Myocardial infarction codes [ICD-10]

Code	Description
I20.0	Unstable angina
I20.1	Angina pectoris with documented spasm
I20.8	Other forms of angina pectoris
I20.9	Angina pectoris, unspecified
I21.01	ST elevation (STEMI) myocardial infarction involving left main coronary artery
I21.02	ST elevation (STEMI) myocardial infarction involving left anterior descending coronary artery
I21.09	ST elevation (STEMI) myocardial infarction involving other coronary artery of anterior wall
I21.11	ST elevation (STEMI) myocardial infarction involving right coronary artery
I21.19	ST elevation (STEMI) myocardial infarction involving other coronary artery of inferior wall
I21.21	ST elevation (STEMI) myocardial infarction involving left circumflex coronary artery
I21.29	ST elevation (STEMI) myocardial infarction involving other sites
I21.3	ST elevation (STEMI) myocardial infarction of unspecified site
I21.4	Non-ST elevation (NSTEMI) myocardial infarction
I21.9	Acute myocardial infarction, unspecified
I21.A1	Myocardial infarction type 2
I21.A9	Other myocardial infarction type
I22.0	Subsequent ST elevation (STEMI) myocardial infarction of anterior wall
I22.1	Subsequent ST elevation (STEMI) myocardial infarction of inferior wall
I22.2	Subsequent non-ST elevation (NSTEMI) myocardial infarction
I22.8	Subsequent ST elevation (STEMI) myocardial infarction of other sites
I22.9	Subsequent ST elevation (STEMI) myocardial infarction of unspecified site

Myocardial infarction codes [ICD-9]

Code	Description
410.00	Acute myocardial infarction of anterolateral wall, episode of care unspecified
410.01	Acute myocardial infarction of anterolateral wall, initial episode of care
410.02	Acute myocardial infarction of anterolateral wall, subsequent episode of care
410.10	Acute myocardial infarction of other anterior wall, episode of care unspecified
410.11	Acute myocardial infarction of other anterior wall, initial episode of care
410.12	Acute myocardial infarction of other anterior wall, subsequent episode of care
410.20	Acute myocardial infarction of inferolateral wall, episode of care unspecified
410.21	Acute myocardial infarction of inferolateral wall, initial episode of care
410.22	Acute myocardial infarction of inferolateral wall, subsequent episode of care
410.30	Acute myocardial infarction of inferoposterior wall, episode of care unspecified
410.31	Acute myocardial infarction of inferoposterior wall, initial episode of care
410.32	Acute myocardial infarction of inferoposterior wall, subsequent episode of care
410.40	Acute myocardial infarction of other inferior wall, episode of care unspecified
410.41	Acute myocardial infarction of other inferior wall, initial episode of care
410.42	Acute myocardial infarction of other inferior wall, subsequent episode of care
410.50	Acute myocardial infarction of other lateral wall, episode of care unspecified
410.51	Acute myocardial infarction of other lateral wall, initial episode of care

410.52	Acute myocardial infarction of other lateral wall, subsequent episode of care
410.60	True posterior wall infarction, episode of care unspecified
410.61	True posterior wall infarction, initial episode of care
410.62	True posterior wall infarction, subsequent episode of care
410.70	Subendocardial infarction, episode of care unspecified
410.71	Subendocardial infarction, initial episode of care
410.72	Subendocardial infarction, subsequent episode of care
410.80	Acute myocardial infarction of other specified sites, episode of care unspecified
410.81	Acute myocardial infarction of other specified sites, initial episode of care
410.82	Acute myocardial infarction of other specified sites, subsequent episode of care
410.90	Acute myocardial infarction of unspecified site, episode of care unspecified
410.91	Acute myocardial infarction of unspecified site, initial episode of care
410.92	Acute myocardial infarction of unspecified site, subsequent episode of care
413.0	Angina decubitus
413.1	Prinzmetal angina
413.9	Other and unspecified angina pectoris

Stroke codes [ICD-10]

Code	Description
G45.0	Vertebro-basilar artery syndrome
G45.1	Carotid artery syndrome (hemispheric)
G45.8	Other transient cerebral ischemic attacks and related syndromes
G45.9	Transient cerebral ischemic attack, unspecified
I61.9	Nontraumatic intracerebral hemorrhage, unspecified
I63.019	Cerebral infarction due to thrombosis of unspecified vertebral artery
I63.119	Cerebral infarction due to embolism of unspecified vertebral artery
I63.139	Cerebral infarction due to embolism of unspecified carotid artery
I63.20	Cerebral infarction due to unspecified occlusion or stenosis of unspecified precerebral arteries
I63.219	Cerebral infarction due to unspecified occlusion or stenosis of unspecified vertebral artery
I63.22	Cerebral infarction due to unspecified occlusion or stenosis of basilar artery
I63.239	Cerebral infarction due to unspecified occlusion or stenosis of unspecified carotid artery
I63.30	Cerebral infarction due to thrombosis of unspecified cerebral artery
I63.40	Cerebral infarction due to embolism of unspecified cerebral artery
I63.50	Cerebral infarction due to unspecified occlusion or stenosis of unspecified cerebral artery
I63.59	Cerebral infarction due to unspecified occlusion or stenosis of other cerebral artery
I65.09	Occlusion and stenosis of unspecified vertebral artery
I65.1	Occlusion and stenosis of basilar artery
I65.29	Occlusion and stenosis of unspecified carotid artery
I65.8	Occlusion and stenosis of other precerebral arteries
I65.9	Occlusion and stenosis of unspecified precerebral artery
I66.09	Occlusion and stenosis of unspecified middle cerebral artery
I66.19	Occlusion and stenosis of unspecified anterior cerebral artery
I66.29	Occlusion and stenosis of unspecified posterior cerebral artery
I66.9	Occlusion and stenosis of unspecified cerebral artery
I67.848	Other cerebrovascular vasospasm and vasoconstriction

Stroke codes [ICD-9]

Code	Description
431	Intracerebral hemorrhage
433.00	Occlusion and stenosis of basilar artery without mention of cerebral infarction
433.01	Occlusion and stenosis of basilar artery with cerebral infarction
433.10	Occlusion and stenosis of carotid artery without mention of cerebral infarction
433.11	Occlusion and stenosis of carotid artery with cerebral infarction
433.20	Occlusion and stenosis of vertebral artery without mention of cerebral infarction
433.21	Occlusion and stenosis of vertebral artery with cerebral infarction
433.30	Occlusion and stenosis of multiple and bilateral precerebral arteries without mention of cerebral infarction
433.31	Occlusion and stenosis of multiple and bilateral precerebral arteries with cerebral infarction
433.80	Occlusion and stenosis of other specified precerebral artery without mention of cerebral infarction
433.81	Occlusion and stenosis of other specified precerebral artery with cerebral infarction
433.90	Occlusion and stenosis of unspecified precerebral artery without mention of cerebral infarction
433.91	Occlusion and stenosis of unspecified precerebral artery with cerebral infarction
434.00	Cerebral thrombosis without mention of cerebral infarction
434.01	Cerebral thrombosis with cerebral infarction
434.10	Cerebral embolism without mention of cerebral infarction

434.11	Cerebral embolism with cerebral infarction
434.90	Cerebral artery occlusion, unspecified without mention of cerebral infarction
434.91	Cerebral artery occlusion, unspecified with cerebral infarction
435.0	Basilar artery syndrome
435.1	Vertebral artery syndrome
435.2	Subclavian steal syndrome
435.3	Vertebrobasilar artery syndrome
435.8	Other specified transient cerebral ischemias
435.9	Unspecified transient cerebral ischemia

Chronic kidney disease codes [ICD-10]

Codes for CKD, hypertensive CKD, and diabetes with CKD

Code	Description
E08.22	Diabetes mellitus due to underlying condition with diabetic chronic kidney disease
E09.22	Drug or chemical induced diabetes mellitus with diabetic chronic kidney disease
E10.22	Type 1 diabetes mellitus with diabetic chronic kidney disease
E11.22	Type 2 diabetes mellitus with diabetic chronic kidney disease
E13.22	Other specified diabetes mellitus with diabetic chronic kidney disease
I12.0	Hypertensive chronic kidney disease with stage 5 chronic kidney disease or end stage renal disease
I12.9	Hypertensive chronic kidney disease with stage 1 through stage 4 chronic kidney disease, or unspecified chronic kidney disease
I13.0	Hypertensive heart and chronic kidney disease with heart failure and stage 1 through stage 4 chronic kidney disease, or unspecified chronic kidney disease
I13.10	Hypertensive heart and chronic kidney disease without heart failure, with stage 1 through stage 4 chronic kidney disease, or unspecified chronic kidney disease
I13.11	Hypertensive heart and chronic kidney disease without heart failure, with stage 5 chronic kidney disease, or end stage renal disease
I13.2	Hypertensive heart and chronic kidney disease with heart failure and with stage 5 chronic kidney disease, or end stage renal disease
N18.1	Chronic kidney disease, stage 1
N18.2	Chronic kidney disease, stage 2 (mild)
N18.3	Chronic kidney disease, stage 3 (moderate)
N18.4	Chronic kidney disease, stage 4 (severe)
N18.5	Chronic kidney disease, stage 5
N18.6	End stage renal disease
N18.9	Chronic kidney disease, unspecified
N19	Unspecified kidney failure

Chronic kidney disease codes [ICD-9]

Codes for CKD, hypertensive CKD, and diabetes with CKD

Code	Description
249.40	Secondary diabetes mellitus with renal manifestations, not stated as uncontrolled, or unspecified
250.40	Diabetes with renal manifestations, type II or unspecified type, not stated as uncontrolled
250.41	Diabetes with renal manifestations, type I [juvenile type], not stated as uncontrolled
403.00	Hypertensive chronic kidney disease, malignant, with chronic kidney disease stage I through stage IV, or unspecified
403.01	Hypertensive chronic kidney disease, malignant, with chronic kidney disease stage V or end stage renal disease
403.10	Hypertensive chronic kidney disease, benign, with chronic kidney disease stage I through stage IV, or unspecified
403.11	Hypertensive chronic kidney disease, benign, with chronic kidney disease stage V or end stage renal disease
403.90	Hypertensive chronic kidney disease, unspecified, with chronic kidney disease stage I through stage IV, or unspecified
403.91	Hypertensive chronic kidney disease, unspecified, with chronic kidney disease stage V or end stage renal disease
404.00	Hypertensive heart and chronic kidney disease, malignant, without heart failure and with chronic kidney disease stage I through stage IV, or unspecified
404.01	Hypertensive heart and chronic kidney disease, malignant, with heart failure and with chronic kidney disease stage I through stage IV, or unspecified
404.02	Hypertensive heart and chronic kidney disease, malignant, without heart failure and with chronic kidney disease stage V or end stage renal disease
404.03	Hypertensive heart and chronic kidney disease, malignant, with heart failure and with chronic kidney disease stage V or end stage renal disease
404.10	Hypertensive heart and chronic kidney disease, benign, without heart failure and with chronic kidney disease stage I through stage IV, or unspecified
404.11	Hypertensive heart and chronic kidney disease, benign, with heart failure and with chronic kidney disease stage I through stage IV, or unspecified
404.12	Hypertensive heart and chronic kidney disease, benign, without heart failure and with chronic kidney disease stage V or end stage renal disease
404.13	Hypertensive heart and chronic kidney disease, benign, with heart failure and chronic kidney disease stage V or end stage renal disease
404.90	Hypertensive heart and chronic kidney disease, unspecified, without heart failure and with chronic kidney disease stage I through stage IV, or unspecified
404.91	Hypertensive heart and chronic kidney disease, unspecified, with heart failure and with chronic kidney disease stage I through stage IV, or unspecified
404.92	Hypertensive heart and chronic kidney disease, unspecified, without heart failure and with chronic kidney disease stage V or end stage renal disease
404.93	Hypertensive heart and chronic kidney disease, unspecified, with heart failure and chronic kidney disease stage V or end stage renal disease
581.81	Nephrotic syndrome in diseases classified elsewhere
583.81	Nephritis and nephropathy, not specified as acute or chronic, in diseases classified elsewhere
585.1	Chronic kidney disease, Stage I
585.2	Chronic kidney disease, Stage II (mild)
585.3	Chronic kidney disease, Stage III (moderate)
585.4	Chronic kidney disease, Stage IV (severe)
585.5	Chronic kidney disease, Stage V
585.6	End stage renal disease
585.9	Chronic kidney disease, unspecified
586	Renal failure, unspecified
